# Does Cancer of Unknown Primary (CUP) Truly Exist as a Distinct Cancer Entity?

**DOI:** 10.3389/fonc.2019.00402

**Published:** 2019-05-17

**Authors:** Tilmann Bochtler, Alwin Krämer

**Affiliations:** ^1^Clinical Cooperation Unit Molecular Hematology/Oncology, Department of Internal Medicine V, German Cancer Research Center, University Hospital Heidelberg, Heidelberg, Germany; ^2^Department of Thoracic Oncology, Thoraxklinik at Heidelberg University Hospital, Heidelberg, Germany; ^3^Department of Internal Medicine V, University Hospital Heidelberg, Heidelberg, Germany; ^4^Translational Lung Research Center Heidelberg (TLRC-H), member of the German Center for Lung Research (DZL), Heidelberg, Germany

**Keywords:** Cancer of unknown primary (CUP), metastasis, classifier assay, chromosomal instability (CIN), next generation sequencing, clonal relationship, clinical trial, treatment

## Abstract

Cancer of unknown primary (CUP) designates an enigmatic cancer entity with histologic confirmation of malignancy from a metastasis but no identifiable primary tumor in spite of a thorough diagnostic work-up. In this review, we discuss the validity of CUP as a distinct cancer entity as well as diagnostic pitfalls. As arguments against a distinct entity, the diagnosis of CUP is erroneous in some cases. Diagnostic pitfalls include incomplete diagnostics, uncertainty in classifying a lesion as either primary or metastasis and mistaking a relapse of an antecedent malignancy as CUP due to histologic and immunohistologic disparities. Given the high frequency of prior malignancies in CUP patients, relapse of an antecedent cancer should always be carefully excluded. Gene expression profiling-based classifier assays aim at aligning the molecular profile of CUP patients with established primary cancer patterns for highest congruency in order to identify the putative primary and treat accordingly. However, the spectrum of predicted putative primaries by molecular techniques is somewhat at odds with the primaries identified in autopsy series. Also, a first randomized clinical trial did not show superiority of primary-tailored therapy over unspecific platinum-based chemotherapy. CUP cases share an aggressive clinical course, atypical metastasis pattern, rapid progression of metastases, a generally poor response to chemotherapy and dismal outcome as distinct clinical features. Metastatic spread appears to take place in the early stages of tumor evolution, with CUP metastases subsequently undergoing genetic evolution toward a chromosomally highly complex and instable karyotype independent from the primary tumor. In clinical practice, the diagnosis of CUP is valid when no primary tumor is detectable. Treatment should ideally offer broad spectrum coverage across numerous malignancies and be well-established in CUP as is the case for carboplatin/paclitaxel and cisplatin / gemcitabine in particular, but it should also cover the most likely putative primary. The diligent diagnosis of CUP is warranted for clinical trials, making the eligibility process particularly laborious. In conclusion, we deem CUP a distinct cancer entity and the diagnosis accurate in most patient cases.

## Introduction

Cancer of unknown primary (CUP) is an enigmatic cancer entity. It is diagnosed in malignancies, where metastases have been histologically confirmed, but where no primary site can be identified in spite of a comprehensive diagnostic work-up (1–4). When making the diagnosis, oncologists frequently meet with incomprehension of patients and relatives, for whom a diagnosis of CUP is hard to accept. Possible theoretical explanation models for the CUP phenomenon like smallness of the primary tumor that evades detection (5) or biological differences between primary and metastases, leading to the regression of the former and expansion of the latter are elusive and hard to grasp. The failure to identify the primary tumor also often makes patients question the diagnosis of malignancy *per se* and coping with the cancer diagnosis even more difficult. It often fosters lingering resentment against chemotherapy, which is unavoidably empiric given the failure to detect the primary. Honestly, CUP specialists do not fare better with some of their fellow oncologists (to say nothing of pathologists), who taunt them that CUP has ceased to exist as a valid diagnosis in the era of molecular diagnostics. In this review, we therefore aim to put the spotlight on the accuracy of CUP diagnosis and the validity of CUP as a distinct cancer entity.

## Arguments Against CUP as a Distinct Cancer Entity

### False or Premature Diagnoses of CUP

All oncologists in the field, who get CUP patients referred from local hospitals, are aware that some diagnoses of CUP are premature or even outright erroneous. Keeping obvious misdiagnoses aside, where histologic confirmation of imaging findings suspicious of a primary tumor is missed, there are also cases with an incomplete diagnostic work-up. Typically, the mandatory diagnostic standard as laid down in the European Society of Medical Oncology (ESMO) guidelines (6) is routinely performed: this includes a histology and meticulous immunohistochemistry, a thorough physical examination, basic blood, and biochemistry analyses as well as CT or MRI imaging of the chest, abdomen and pelvis. However, further tests as determined by clinical judgement based on the clinical picture and the immunohistologic profile are sometimes left out. From our own experience with patients presenting for second opinion at our center, diagnostic efforts among centers and patients differ widely. While the bare minimum of tests as required by the ESMO guidelines has been performed in some patients, many patients have received an extensive diagnostic work-up far beyond the requirements of the ESMO guidelines. Thus lacking or insufficient diagnostic tests might lead to an erroneous diagnosis only in some patients. The correct diagnosis of CUP also strongly relies on the clinical judgement and experience of the treating oncologist.

At least at our center, we repeatedly observe delicate cases where a relapse of a prior malignancy has to be considered as a differential diagnosis to a new CUP. Some patients referred as CUP in truth suffer instead from relapse of an antecedent malignancy which was disregarded due to histologic or immunohistologic disparities between the two tumors. Given a high rate of prior malignancies of around 20–25% among CUP patients (7), the identification of cryptic relapses of an antecedent malignancy is highly relevant in many patients. In 11 cases, we were skeptical of the CUP diagnosis and considered relapse of the antecedent malignancy as a differential diagnosis (7). We addressed these questionable cases by comparative panel sequencing of both tumors to elucidate their clonal relationship. Based on fully or largely overlapping mutational spectra, seven out of 11 presumed CUP cases could be reclassified as relapses of the known antecedent malignancy, whereas largely divergent mutational patterns established clonal independence of the tumors in four out of 11 cases and thus corroborated the diagnosis of CUP. Interestingly, all of these four patients harbored a germline mutation which might have played a predisposing role in both cancers. Markedly, all patients with overlooked relapse of a prior malignancy had been scheduled with our CUP clinic, thus excluding clinically obvious relapse cases. It should also be noted that in these cases histologies of the antecedent cancer and the proposed CUP were widely different up to situations where an adenocarcinoma in one and a squamous cell carcinoma in the other sample was found. This study strongly cautions against a premature diagnosis of CUP in patients with antecedent malignancies (7). At our center, we now have adopted a policy of parallel comparative panel sequencing of both prior malignancy and CUP tumor to elucidate their clonal relationship in dubious cases. Obviously, we cannot rule out a polyclonal cancer origin as a potential pitfall when assessing cancer relationships with this comparative molecular panel sequencing approach (8).

Another delicate aspect is the clinical judgement whether a malignant lesion should be classified as primary cancer or metastatic site, thus entailing the diagnosis of CUP. A typical example is a patient with isolated CK7+ adenocarcinoma metastases of the liver, where cholangiocellular carcinoma (CCC) has to be considered as a differential diagnosis to CUP with hepatic metastases. Likewise, a CK7+, TTF1- adenocarcinoma lung mass might represent a primary lung cancer or alternatively a CUP with pulmonary metastasis.

### CUP With Primary Tumor Unmasked During Disease Course or at Surgery or Autopsy

In some CUP patients, the primary tumor unmasks itself over time during the disease course, or is finally detected at autopsy, ultimately leading to a revision of the CUP diagnosis. The emergence of the primary tumor during the lifetime of the patient is rare with frequencies in the 20% range reported in the literature (5) and even less frequent in our experience. In contrast, the rate of detection of the primary tumor is much higher in autopsy series, which finally demonstrate a primary in as many as 50–80% of CUP cases, leading to a posthumous revision of the CUP diagnosis (9–15). By autopsy, lung, large bowel and pancreas cancers appear as the prevailing underlying primary cancers.

### Hints Toward the Likely Primary by Molecular Profiling

**Figure 1:**
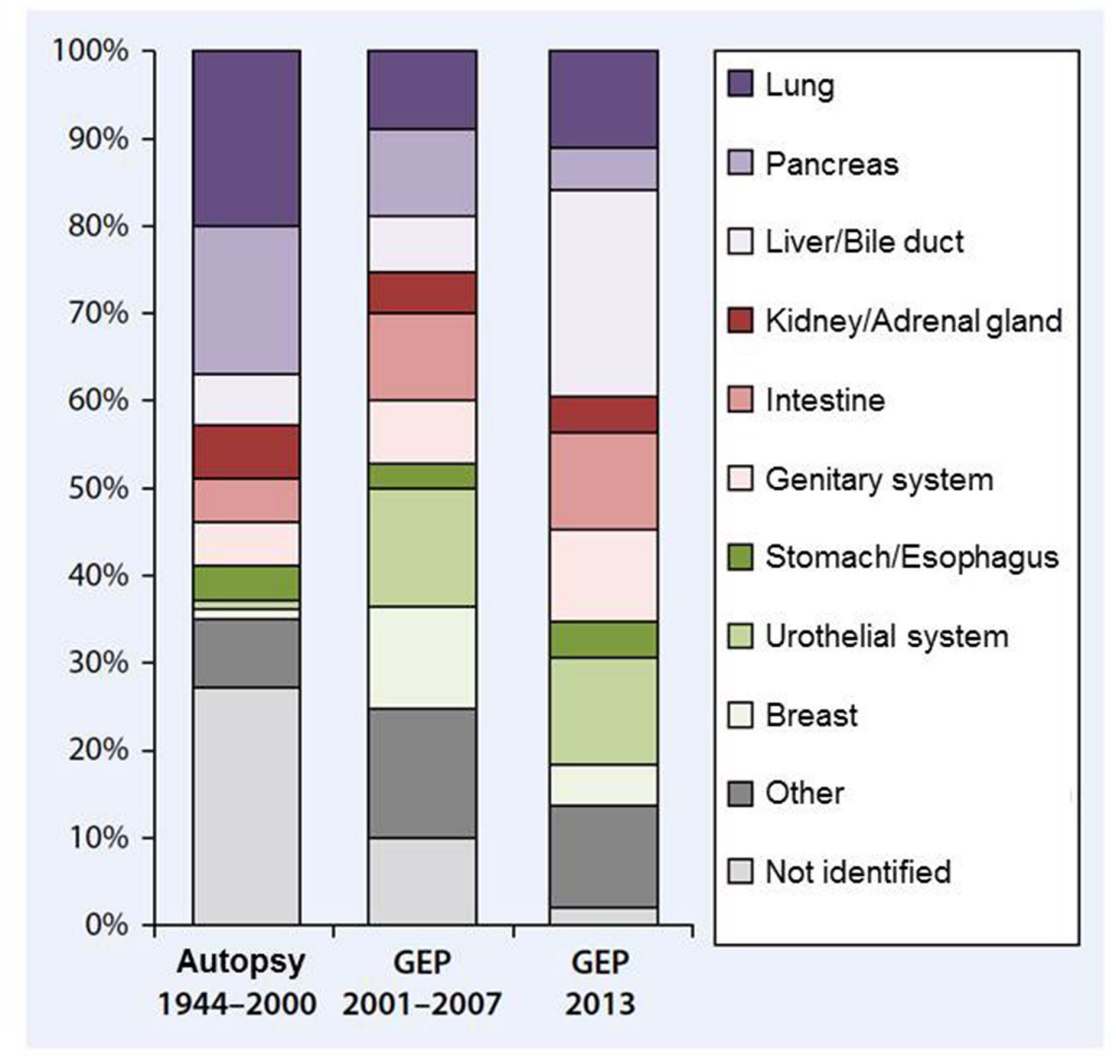
displays the relative frequencies of (presumed) primary sites in CUP cancers. Pooled data from 12 autopsy studies including 844 autopsies from 1944-2000 (left) are juxtaposed to gene expression profiling data from more than 500 patients drawn from four studies published from 2001 to 2007 (middle) and 252 patients published in 2013 (right), drawn from Loffler et al. (18), previously adapted from (9, 19). Unfortunately, no autopsy and molecular data are available from the same decade, so the discrepant putative primary frequencies between the autopsy and the molecular studies might either reflect inconsistencies between both approaches or alternatively a bias by time decade.

Advances in molecular diagnostics and microarray technology have raised expectations that molecular profiling might provide hints toward the likely primary tumors. For that purpose gene expression profiling-based classifier assays have been brought forward in CUP, which aim to align the respective profile of a particular CUP case with the best match from previously established profile databases from all sorts of cancer entities. Hereby, the assignment of molecular signatures to cancers is typically developed and validated in cancers with known primary before the molecular profile of a CUP tumor is aligned to the established primary pattern with the highest congruency (9). These classifier assays can successfully identify the primary in 76–96% of cancer cases with known primary and predict the likely primary tumor in 83–90% of CUP specimens, assuming that the CUP cancer has retained the basic gene expression signature of the tissue of origin during metastatic spread (5, 9, 16, 17). However, doubts remain about the reliability of this approach. It lies in the very nature of CUP that no definitive verification of the molecular classification is at hand (9). Furthermore, the spectrum and respective frequencies of molecularly identified likely primary cancers differ from those in autopsy series (9–15), with breast, urothelial and colorectal primaries overrepresented in the former and pancreas and lung cancers predominating in the latter (Figure 1) (9). However, as a caveat the respective autopsy and molecular studies come from different decades, allowing for a time bias in case the spectrum of putative primaries might have changed over the last two decades.

Current studies are investigating response to treatment tailored to the primary predicted by classifier assays as proof-of-principle trials. In one small study in 45 CUP patients the identification of a putative primary tumor routinely treated with carboplatin/paclitaxel indeed predicted an actual response to this regimen (20). In a larger study by Hainsworth and coworkers CUP patients were scheduled to receive site-specific treatment based on the molecular gene expression classifier essay. In this non-randomized trial the two thirds of patients actually receiving assay directed therapy reached a median overall survival of 12.5 months, which compares favorably with historic cohorts (19). Markedly, within the study cohort patients predicted to suffer from treatment responsive cancers indeed displayed an improved prognosis. Accordingly, Hainsworth and Greco have concluded that the paradigm change toward a molecular work-up has become clinical reality (21). However, in a large randomized phase II trial site-specific therapy based on comprehensive gene expression profiling did not improve prognosis as compared to empirical carboplatin/paclitaxel chemotherapy in the comparator arm, with median overall survival times of 9.8 vs. 12.5 months, respectively (22).

In addition to gene expression profiling, Moran and coworkers have employed epigenetic profiling by DNA methylation profiles in the search of the presumed primary (23). In their comprehensive approach, the experience gained from methylation profiling in large training and validation sets from cancer patients with known primary was used to classify 216 CUP cases. Subsequent clinical detection of the primary during further follow-up as well as histology and immunohistology were used to countercheck the accuracy of this approach that identified the putative cancer of origin in 188/216 (87%) of patients. Patients who received a chemotherapy regimen tailored to the putative primary based on clinical and pathological findings achieved improved overall survival, although molecular profiling was performed only retrospectively. This study might advocate more site-directed chemotherapies at least in cases where clinical and/or pathological findings are suggestive of a specific site. Together with the studies by Hainsworth et al. (19) and Hayashi et al. (22) it remains debatable whether site-specific chemotherapy tailored to the putative primary is beneficial. Furthermore, as also discussed by Moran et al. (23), a CUP tumor identified by DNA methylation profiling might still be biologically distinct from its metastatic equivalent with a known primary tumor.

**Figure 2:**
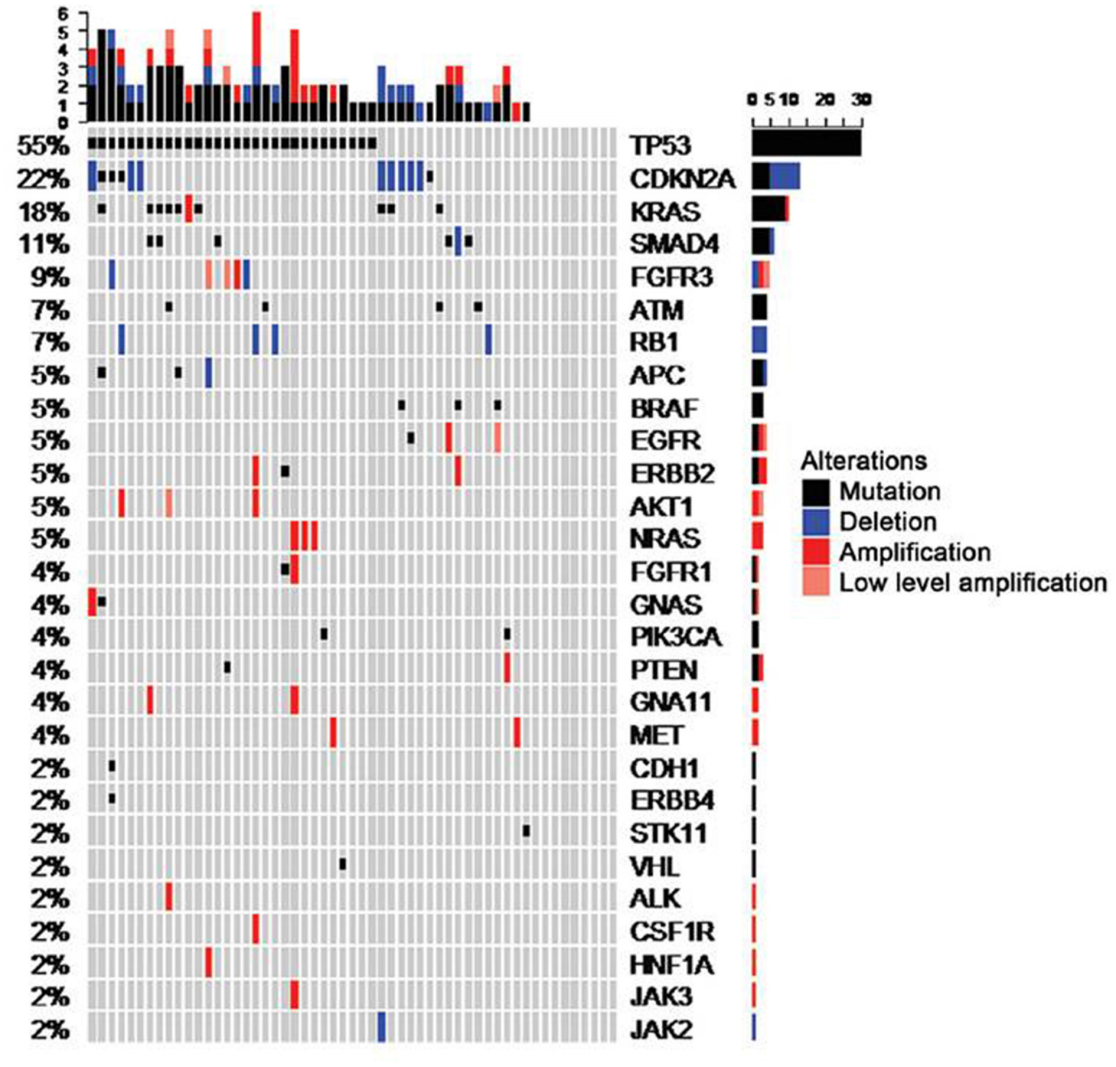
shows the distribution of mutational spectra in CUP patients with adenocarcinoma and undifferentiated carcinoma types as detected in a prior study from our group, drawn from Loffler et al. (26).

Whereas the classifier assays discussed above fully focus on hints toward the primary tumor, panel sequencing strategies aim at identifying targets for molecularly driven therapies independent of the tissue of origin instead (24–27). Accordingly, the mutational spectra in these cases usually do not give substantial hints toward the most likely primary. For example, mutations of *TP53* are by far the most abundant mutations found in CUP cancers (Figure 2), but this mutation is recurrently found throughout almost any kind of malignancies, thus precluding putative site allocation. Mutations providing definitive hints toward the most likely primary like *ALK* translocations for lung cancer are scarce in CUP.

## Arguments in Favor of CUP as a Distinct Cancer Entity

### Thoroughness of Clinical Work-Up

As discussed above at least in the German health care system many patients have received an extensive diagnostic work-up far in excess of the minimum requirements of the ESMO guidelines (6). Gastroscopies and colonoscopies are almost routinely performed in every single patient. The diagnostic work-up also often includes PET-CT scans for CUP types where they are not even recommended as standard of care in the ESMO guidelines. In spite of these diagnostic efforts, the primary cancer is not identified in most cases. From a clinical perspective, the diagnosis of CUP is therefore confirmed even after an exhaustive diagnostic work-up in most cases, and false diagnosis of CUP due to lacking or insufficient diagnostic tests appears as a rare phenomenon. Admittedly, the interpretation of results is a tricky and error-prone process.

### Distinct Clinical Features Inherent to CUP

As reviewed by Pentheroudakis et al. (9) CUP cases typically share distinct clinical features: an aggressive clinical course, atypical metastasis pattern and rapid progression of metastases, a generally poor response to chemotherapy and dismal patient outcome, with overall survival rates in the 1 year range even in clinical trial cohorts (28–33). Whereas the primary tumor remains clinically insignificant during the clinical course, the disease is characterized by early and rapidly progressing metastatic spread. Therefore, an underlying CUP-specific “pro-metastatic” genetic signature has been postulated. In tune with this concept, the prognosis of CUP patients is inferior to the prognosis of cancer patients with distant metastases from a known primary, both in general and when CUP cases with a presumed primary are compared to the “equivalent” metastatic disease with known primary (5, 9). Furthermore, the pattern of metastatic spread is intriguingly distinct in CUP cases, with a high frequency of lung, brain and bone metastases as well as unusual metastatic sites (9). Therefore, according to this model CUP cases appear to display a distinct natural history and biological properties rather than being an accumulation of diverse cancers which merely share the failure of an identifiable primary tumor (5).

New insights into the mechanisms of metastatic seed in cancers also provide explanatory models for the enigmatic phenomenon of an undetectable primary tumor. Experimental data from mammary cancer animal models imply that metastatic spread takes place in the early stages of tumor evolution (34, 35). In these models invasive early tumor cells, which are genetically less evolved and display stemness features, are capable to migrate in the blood stream as disseminated cancer cells (DCC) and found metastases far before an overt primary cancer can be found. Apparently, these early DCCs have the potential to switch between migration, dormancy and proliferation programs. Once proliferation sets in, the tendency to disseminate appears to decline. This concept of early dissemination of tumor cells implies subsequent independent progression of primary tumor and metastases, which both grow under the selection pressure of the immune system and the respective microenvironment (36). This model of early branching of the primary tumor and metastases evolution and their long independent trajectory under selection pressure in different niches obviously accounts for genetic and growth discrepancies between primary and metastases. Seen from this angle, CUP can be regarded as the extreme end of an independent parallel evolution where metastases have largely outgrown the primary tumor. From this perspective, it can also be speculated that CUPs share a prominent early DCC phase as a unifying biologic feature.

Indeed, some findings in CUP seem to support the validity of this model, including the clinically observed high systemic relapse rate in CUP patients with localized disease treated by surgery and / or radiotherapy in curative intent (37). Cytogenetic data support this model as well (38). In a study by Pantou and coworkers CUP tumors were shown to display advanced cytogenetic patterns, with abnormal karyotypes harboring numerous, complex and unbalanced cytogenetic aberrations. An average of 15 chromosomal aberrations was found per case, increasing to 22.6 when ploidy changes were considered as well, which is well in excess of metastatic disease with known primary (38). In view of chromosomal instability (CIN) as a driver of tumor evolution this karyotypic complexity in CUP reflects the aggressiveness of metastatic growth in this entity. Markedly, within the study cohort the patients with massive chromosomal changes had an even worse prognosis (38). These data were fully corroborated by a study by Vikesa and coworkers, who also found a high level of CIN in CUP and a correlation of karyotypic complexity with dismal prognosis. Interestingly, alignment of the cytogenetic profiles of CUP patients with the respective profiles from known cancer entities in this study showed that CUPs were more distantly related to the predefined tumor classes than metastases from known primaries. Interestingly, this equally applied to CUP cases with the primary cancer identified or still elusive during the further clinical course (39). Accordingly, the authors concluded firstly that CUP exhibits distinct molecular features, and secondly that CIN facilitates primary tumor independent progression of metastatic sites in CUP following early dissemination and leading to poor outcome (39).

Spontaneous tumor regressions have been reported throughout a variety of malignancies, which are attributed to tumor cell elimination by the immune system in view of the frequent association with infections observed in these cases. The concept of immune-mediated cancer surveillance is further supported by an increased cancer incidence in transplant recipients on immunosuppressants and the recent success of immune checkpoint inhibitors in numerous cancer entities. Thus, it is possible that the lack of a primary tumor in CUP is an immune mediated event at least in some cases as well. Recently, it was demonstrated across several cancer entities that a high degree of CIN confers resistance to immune mediated therapies (40). Therefore, it can be speculated that the particularly high degree of CIN in CUP metastatic sites makes CUP tumors resistant to immune surveillance, whereas the corresponding less chromosomally instable primary tumors have regressed.

Interestingly, evidence suggests that primary tumors in general actively modify future metastatic sites by tumor-secreted factors to make them susceptible to metastatic seed, a phenomenon called “premetastatic niche formation” (41, 42). It could be hypothesized that in CUP cases seeding tumor cells are sufficiently aggressive themselves, allowing for metastasis formation independent from this facilitation by the primary tumor.

### Limitations of Molecular Profiling

In spite of unquestionable progress, mutational profiling of CUP has its limitations. Nowadays, panel sequencing is increasingly performed on a routine basis (24–27, 43). However, as discussed above the mutational profile obtained by these panel sequencing approaches typically does not permit to draw conclusions regarding the tissue of origin. In CUP, *TP53* mutations are by far most abundant (Figure 2). Given that *TP53* mutations are found throughout all types of carcinomas this does not permit conclusions regarding a putative primary site. Likewise, other frequent mutations in CUP including *RAS, CDKN2A, MYC, ARID1A, PIK3CA*, or *BRAF* are not tissue specific.

The classifier assays discussed above, although designed to detect the putative primary, have not established themselves in clinical routine so far. None of these tests is either marketed or insurance-covered in Germany nor are these procedures recommended in the ESMO clinical guidelines for CUP diagnostics and treatment (6).

Last but not least, no molecular profiling test can substitute for a clinically identified primary tumor. Even putting aside the disquieting frequency of discrepancies in the distribution of primary sites between autopsy and molecular profiling patient series (Figure 1), a CUP tumor with a distinct molecular profile suggestive of a primary cancer behaves biologically most likely still different from the respective primary cancer. Therefore, in the final conclusion of two insightful reviews, Pentheroudakis et al. judge that in view of lacking proof of prognostic benefit and of methodological uncertainties, molecular profiling has not (yet) become the benchmark for CUP primary detection (9, 44).

## Clinical Reality—Valid Diagnosis of CUP and Justified Assumption of a Putative Primary Tumor

In most cases, the CUP diagnosis is correct, because metastatic spread has been histologically confirmed and a primary tumor has remained elusive in spite of a thorough work-up according to the ESMO guidelines (6), thereby meeting the criteria how CUP is defined. At the same time, in many patients the clinical picture along with the histologic, immunohistologic and molecular profile is suggestive of a putative primary. Nevertheless, the diagnosis of CUP remains valid as long as no primary tumor in the respective organ is detectable.

For reasons described above, in cases with a putative primary, treatment should follow the treatment algorithms for the suspected primary cancer. For example, if a patient is diagnosed with a CK20+, CDX2+, CK7– adenocarcinoma with liver and peritoneal metastases, both the immunohistologic profile and the distribution of metastatic sites is in tune with colorectal cancer, and treatment should be administered according to protocols for metastatic colorectal cancer (45–47). Likewise, a patient with squamous cell carcinoma of cervical lymph nodes probably suffers from head and neck cancer and should be treated accordingly (48–50). These two distinct clinical constellations highly suggestive of a putative primary and requiring specific site-directed therapy are—along with others—accounted for in the ESMO CUP guidelines as specifically defined favorable subsets (6). Even in entities not listed as distinct favorable subtypes in the ESMO classification, as is the case for CK7+ TTF1+ carcinomas in patients with mediastinal or hilar lymph nodes or pleural carcinosis, the treatment should be dictated by the most likely primary, in this case lung cancer. Even in cases where the clinical suspicion is less clear-cut – for example in cases of an immunohistologic profile suggestive of gastrointestinal cancer, many oncologists would prefer a gastrointestinal protocol like FOLFOX, FLO or FLOT over empiric standard CUP chemotherapy with carboplatin / paclitaxel or cisplatin/gemcitabine (30, 32, 33, 51). In conclusion, the treatment should ideally offer broad spectrum coverage across numerous malignancies and be well-established in CUP as is the case for carboplatin/paclitaxel and cisplatin/gemcitabine in particular, but it should also cover the most likely putative primary. Obviously, in sophisticated cases pros and cons must be deliberated and decisions will also depend on the preferences of the treating oncologist. Nevertheless, even when oncologists deem circumstantial evidence sufficient to recommend treatment tailored to the putative primary, the diagnosis of CUP is still valid as long as the primary tumor cannot be nailed down. We are aware that there is a twilight zone between circumstantial hints pointing toward a putative primary but still compatible with the diagnosis of CUP, and unequivocal evidence for the primary tumor.

## Clinical Trials—Importance of Precise CUP Diagnosis and Need for Further Standardization

Data from clinical trials in CUP are scarce (28, 29), with only few phase II studies (30–32, 52–54) and a single phase III study (55). Additionally, these clinical trials struggled with patient recruitment, partly leading to premature study closure prior to the recruitment of the targeted patient number. Also, patient cohorts are heterogeneous and thus not fully comparable, with favorable subtypes included in some studies but not in others. In daily clinical practice, when a primary is not confirmed but clinically likely due to the clinical picture and the immunohistologic profile, it is absolutely sound to make the diagnosis of CUP and to treat the patient tailored to the putative primary. However, it is highly questionable whether such patients should be eligible for a clinical CUP trial. This concern applies in particular to cases where the standard empiric CUP chemotherapy regimens (28–32, 53, 54) provided in the respective trials do not fully match with the treatment required for the likely primary.

There is broad consensus that the ESMO guidelines (6) should be applied for clinical trials. However, they leave room for interpretation. Consensus guidelines for clinical trials in CUP have not been defined so far, and likely would also not be able to unequivocally define the “typical” picture of unfavorable CUP as target population for clinical trials. Nevertheless, we feel that a meticulous check of clinical cases by the sponsor is required at study inclusion, since the quality of a clinical trial in CUP also hinges on the inclusion of “true” CUP patients.

## Conclusions

CUP cases are biologically characterized by early and aggressive metastatic spread, poor response to chemotherapy and poor prognosis, which has led to the postulation of a unifying underlying pro-metastatic signature in CUP.

In the era of molecular work-up further tools beyond histology and immunohistochemistry have become available to characterize cancers. CUP classifier assays have been developed which determine the putative tissue of origin of a CUP cancer by alignment with molecular profiles established for cancers with known primary. Even if the molecular signature points toward a putative primary tumor, the diagnosis of CUP remains still valid as long as no primary tumor is detectable. However, molecular analysis, immunohistochemistry and clinical picture should weigh in to adjust treatment to the putative primary. It remains at the discretion of the treating physician to weigh clinical, immuno-histochemical, and increasingly molecular findings as well.

Some patients receive a diagnosis of CUP prematurely and the diagnosis should always be questioned by an experienced oncologist. Relapse of a prior malignancy should be meticulously excluded.

Being committed and dedicated to advancing research in the field of CUP, we as authors admit to be biased. Nevertheless, we feel that CUP appears as a valid cancer entity and that most, though not all, patients diagnosed with CUP indeed suffer from a “true” CUP.

## Author Contributions

Both authors listed have made a substantial, direct and intellectual contribution to the work, and approved it for publication.

### Conflict of Interest Statement

Both authors work as study oncologists for the CUPISCO trial, which is sponsored by Roche, and have received reimbursement for study related travels as well as remuneration for their work as study oncologists for the benefit of their employer.

## Abbreviations

CIN: chromosomal instability
CK: cytokeratin
CUP: cancer of unknown primary
DCC: disseminated cancer cell
TTF1: thyroid transcription factor-1.
